# SARS-CoV-2 Inhibitors Identified by Phenotypic Analysis of a Collection of Viral RNA-Binding Molecules

**DOI:** 10.3390/ph15121448

**Published:** 2022-11-22

**Authors:** Alvaro Simba-Lahuasi, Ángel Cantero-Camacho, Romel Rosales, Briana Lynn McGovern, M. Luis Rodríguez, Vicente Marchán, Kris M. White, Adolfo García-Sastre, José Gallego

**Affiliations:** 1 Centro de Investigación Traslacional San Alberto Magno, Universidad Católica de Valencia, 46001 Valencia, Spain; 2 Escuela de Doctorado, Universidad Católica de Valencia, 46001 Valencia, Spain; 3 Department of Microbiology, Icahn School of Medicine at Mount Sinai, New York, NY 10029, USA; 4 Global Health Emerging Pathogens Institute, Icahn School of Medicine at Mount Sinai, New York, NY 10029, USA; 5 Departament de Química Inorgànica i Orgànica, Secció de Química Orgànica, Institut de Biomedicina (IBUB), Universitat de Barcelona (UB), 08028 Barcelona, Spain; 6 Department of Medicine, Division of Infectious Diseases, Icahn School of Medicine at Mount Sinai, New York, NY 10029, USA; 7 Tish Cancer Institute, Icahn School of Medicine at Mount Sinai, New York, NY 10029, USA; 8 Department of Pathology, Molecular and Cell Based Medicine, Icahn School of Medicine at Mount Sinai, New York, NY 10029, USA

**Keywords:** antiviral drug, coronavirus, COVID-19, RNA, SARS-CoV-2, 3’UTR S2m hairpin

## Abstract

Antiviral agents are needed for the treatment of SARS-CoV-2 infections and to control other coronavirus outbreaks that may occur in the future. Here we report the identification and characterization of RNA-binding compounds that inhibit SARS-CoV-2 replication. The compounds were detected by screening a small library of antiviral compounds previously shown to bind HIV-1 or HCV RNA elements with a live-virus cellular assay detecting inhibition of SARS-CoV-2 replication. These experiments allowed detection of eight compounds with promising anti-SARS-CoV-2 activity in the sub-micromolar to micromolar range and wide selectivity indexes. Examination of the mechanism of action of three selected hit compounds excluded action on the entry or egress stages of the virus replication cycle and confirmed recognition by two of the molecules of conserved RNA elements of the SARS-CoV-2 genome, including the highly conserved S2m hairpin located in the 3’-untranslated region of the virus. While further studies are needed to clarify the mechanism of action responsible for antiviral activity, these results facilitate the discovery of RNA-targeted antivirals and provide new chemical scaffolds for developing therapeutic agents against coronaviruses.

## 1. Introduction

The current COVID-19 pandemic has shown the need to develop effective measures to prevent and treat infections by SARS-CoV-2 (SCoV2) and other coronavirus outbreaks that will likely occur in the future. Although effective vaccines have been developed against SCoV2, a large part of the world population remains unvaccinated, and the current antiviral treatments rely on small-molecule compounds targeting viral enzymes or monoclonal antibodies inhibiting virus entry [1]. New agents with alternative mechanisms of action are needed to treat SCoV2-infected individuals and to increase the antiviral armamentarium against coronaviruses.

Among the possible mechanisms for blocking the replication cycle of RNA viruses, strategies based on targeting structured RNA elements have not been exploited to the same extent as other direct-action approaches, mainly due to the challenges associated with specific RNA recognition [2]. However, viral RNA transcripts dominate the transcriptome of SCoV2-infected cells, accounting for 65% of sequencing reads [3]. This translates into a high intracellular concentration of SCoV2 sequences, which may facilitate viral RNA targeting. In addition, functional RNA structures are conserved across different coronaviruses [4], making drugs directed against these motifs more likely useful to control future outbreaks. These considerations encourage RNA-targeting antiviral research.

**Figure 1:**
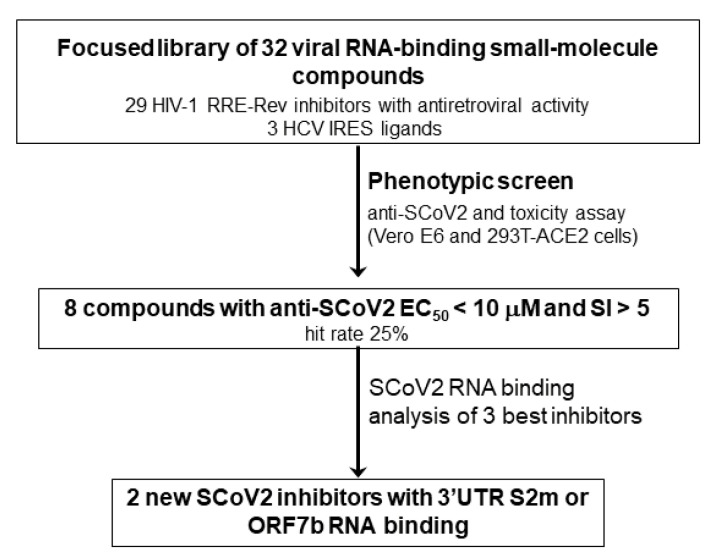
Phenotypic strategy used for identifying RNA-binding small molecules with anti-SCoV2 activity.

In the context of the ongoing pandemic, we carried out a phenotypic study to rapidly detect possible RNA-binding molecules with antiviral action (Figure 1). This was accomplished by examining a small library of compounds previously shown to have viral RNA-binding properties. The screen was carried out using a live-virus SCoV2 antiviral assay with concurrent cellular toxicity measurements and allowed the identification of eight small-molecule agents with significant antiviral activity and selectivity indexes. Cellular time-of-addition experiments involving the FDA-approved drug clomiphene citrate and two drug-like trityl-imidazole and piperidinyl-quinazolinone molecules excluded action on the entry or egress stages of the virus replication cycle. Subsequent NMR spectroscopy and fluorescence experiments revealed recognition by the trityl-imidazole and piperidinyl-quinazolinone compounds of conserved RNA structures of the virus.

## 2. Results

### 2.1. Selection of Compounds for the Phenotypic Screen

In an attempt to identify new SCoV2 inhibitors with a mechanism of action based on RNA-binding, a small library of 32 compounds targeted towards human immunodeficiency virus type-1 (HIV-1) or hepatitis C virus (HCV) RNA structures was assembled (Table S1). The HIV-1 subset contained 29 molecules experimentally shown to bind subdomain IIB of the rev recognition element (RRE) of the HIV-1 genomic RNA. This subset included five FDA-approved drugs and six drug-like molecules identified through experimental screens based on monitoring the interaction between RRE and the virus-encoded protein Rev [5,6], five analogs of the FDA-approved RRE-Rev inhibitor clomiphene, four drug-like compounds identified through virtual pharmacophore searches based on the structure of bioavailable RRE-Rev inhibitors [7], and nine terphenylene mimics of the RNA-binding α-helix of Rev [8,9]. The majority (83%) of the compounds in this subset exhibited significant antiretroviral activity in infection assays, and in many cases, this activity was shown with cell-based experiments to be related to inhibition of HIV-1 RNA biogenesis steps, including blockage of Rev action [5,6,7,8,9]. The HCV subset contained three molecules experimentally shown to recognize subdomain IIa of the internal ribosome entry site (IRES) RNA of HCV; these compounds were identified through pharmacophore virtual searches [10] based on known HCV IRES binders [11]. Excluding chemical analogs, the 32 molecules included in the library comprised 19 unique chemical scaffolds (Table S1).

### 2.2. Anti-SCoV2 Activities and Selectivity Indexes

The activity of the viral RNA-targeted molecules against SCoV2 was evaluated with dose–response live-virus immunofluorescence-based experiments carried out in Vero E6 cells. The hit rate turned out to be substantially high, with 14 of the 32 compounds evaluated (44%) having anti-SCoV2 EC_50_ values below 10 μM (Table S1 and Figure S1). Ignoring chemical analogs and considering unique chemical scaffolds only, the percentage of active molecules increased to 58%.

In order to focus on the best inhibitors, we next confirmed our results in a human cell line, 293T-ACE2. Taking into account the antiviral and toxicity data from both cell types, 8 molecules (25% of all compounds examined) exhibited EC_50_ values below 10 μM in both cell lines together with a selectivity index (SI; calculated as CC_50_/EC_50_) above 5 in at least 1 cellular type. These compounds comprised one drug-like molecule from the HCV IRES subset and seven molecules from the HIV-1 RRE subset, including three clinically used drugs and four drug-like molecules (Table S2 and Figure S2).

**Figure 2:**
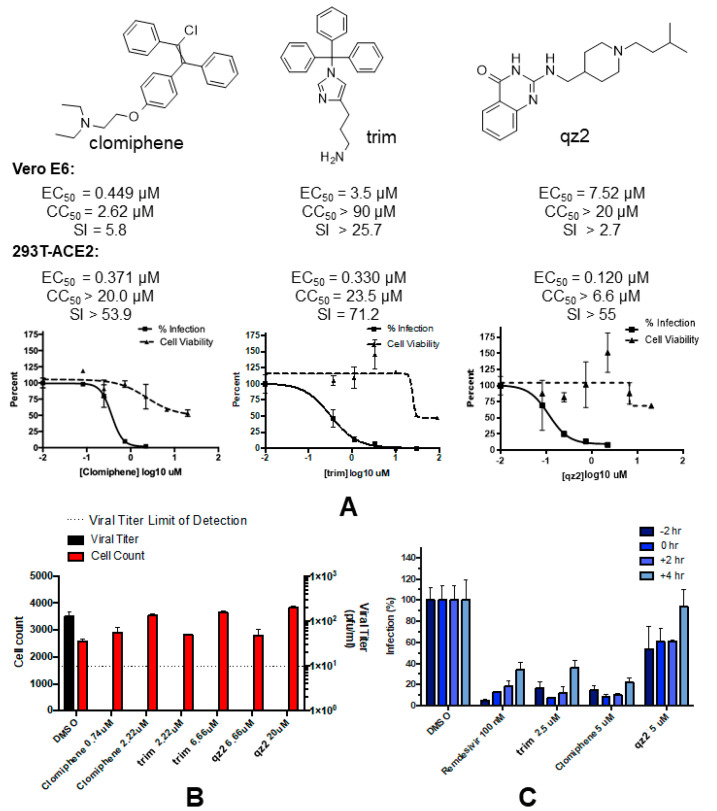
Chemical structure, SCoV2 antiviral activity, and toxicity of the best three RNA-binding SCoV2 inhibitors selected from the phenotypic study. (**A**) Chemical structure and antiviral and toxicity data of clomiphene citrate, trim, and qz2, obtained in Vero E6 and human 293T-ACE2 cells. The dose–response graphs correspond to 293T-ACE2 experiments. (**B**) Virus titers determined in supernatants by TCID_50_ at 48 h post-infection, with DAPI cell count from the well indicated. The limit of detection for viral titers is indicated with a dotted line. (**C**) Time-of-addition assays analyzing the antiviral action of clomiphene citrate, trim, and qz2. The data were normalized to the mean of DMSO-treated cells at each time point, and remdesivir was used as a positive control.

On the basis of antiviral EC_50_ values, selectivity indexes, and repurposing potential, three compounds were selected for further examination: the FDA-approved HIV-1 RRE inhibitor clomiphene citrate, an additional trityl-imidazole RRE inhibitor (hereafter identified as trim), and a piperidinyl-quinazolinone HCV IRES ligand (qz2) (Figure 2A). These molecules exhibited anti-SCoV2 EC_50_ values between 0.120 and 7.52 μM in both cell types, together with selectivity indexes between 2.7 and 71 (Figure 2A). Infectious viral particles were subsequently quantified in the supernatants of SARS-CoV-2 infected cells in the presence and absence of these compounds. Clomiphene citrate, trim, and qz2 were able to reduce viral titers below the limit of detection, from the low but detectable viral titers produced in our low-cell-count antiviral screening assay (Figure 2B). No loss in DAPI cell counts was observed, indicating that the compound concentrations used in the experiments were not toxic. In fact, slight increments in DAPI cell counts were detected with augmenting compound concentrations, which may indicate protection from the virus cytopathic effect. Based on these results, all subsequent experiments focused on these three molecules.

### 2.3. Time-of-Addition Assays

In order to narrow down the location of the block in viral replication caused by the selected compounds in the virus life cycle, we carried out cell infection assays adding the inhibitor at different times during the SCoV2 replication cycle (Figure 2C). In this 8-h single-cycle (MOI = 2) infection experiment, each of the selected molecules was added to 293T-ACE2 cells at −2, 0, +2, or +4 h relative to infection. Remdesivir, a nucleoside analog inhibitor of the viral RNA polymerase, was used as a reference in these experiments. trim, clomiphene, and qz2 strongly inhibited nucleocapsid protein expression even when added at +2 h relative to infection. As observed with remdesivir, this inhibition decreased when adding the molecules at +4 h. Maximum inhibition was observed at 0 and +2 h for trim and clomiphene, and at −2 h for qz2 (Figure 2C). The time-dependent viral inhibition patterns of clomiphene, trim, and qz2 excluded mechanisms of action targeting viral entry, which typically lose the majority antiviral activity by hour 0, as well as those targeting viral egress, which have no antiviral activity in this single-cycle assay measuring viral N protein expression. This leaves the viral replication stage as the most likely target of these compounds.

### 2.4. SCoV2 RNA-Binding Experiments

#### 2.4.1. Selection of RNA Sequences

**Figure 3:**
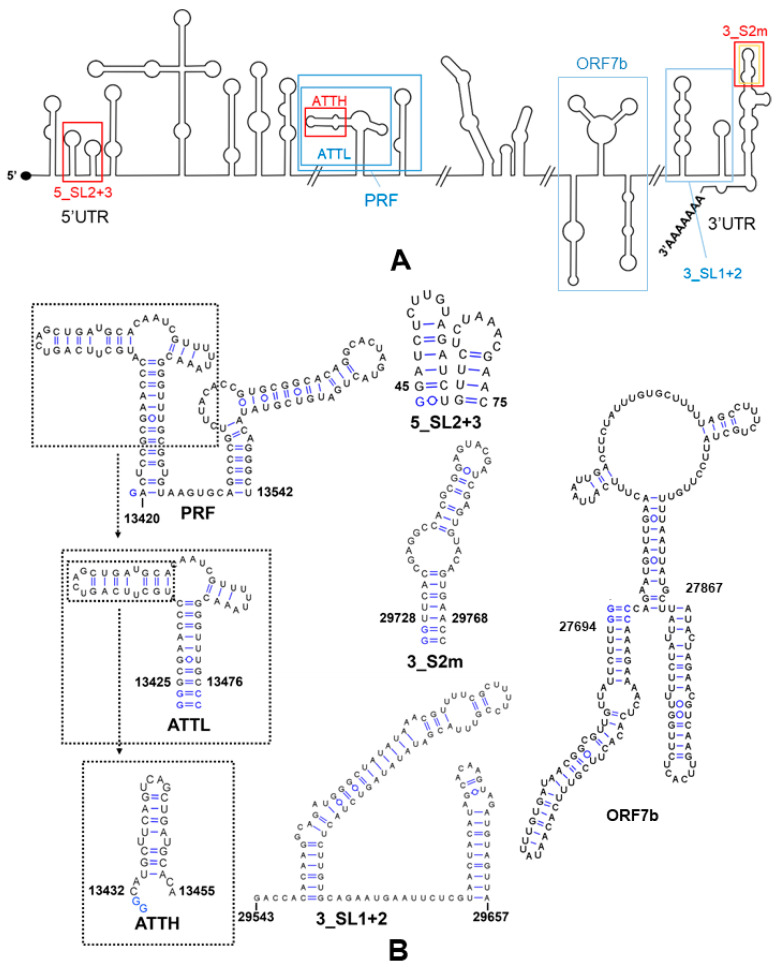
SCoV2 genome and viral RNA elements analyzed in this study. (**A**) Secondary structure representation of the SCoV2 genome, indicating the domains examined as possible targets for clomiphene citrate, trim, and qz2. Blue rectangles indicate secondary structure domains analyzed with ligand-based NMR experiments. Red rectangles mark domains studied with ligand-based and RNA-based NMR experiments. The orange rectangle around the 3_S2m hairpin indicates that this domain was additionally analyzed with fluorescence experiments. The small ATTH domain was investigated with RNA-based NMR experiments only. (**B**) Secondary structure of the SCoV2 RNA constructs evaluated in this report: 32-nt 5_SL2+3 domain, 124-nt full-length PRF domain, 56-nt PRF ATTL subdomain, 26-nt PRF ATTH hairpin, 176-nt ORF7b segment, 115-nt 3_SL1+2 domain, and 45-nt 3_SL2m stem–loop. The blue-colored nt mark changes relative to the wild-type sequence introduced to increase transcription yield. The viral nt encompassed by each domain are indicated using genomic numbering.

In order to evaluate whether the selected compounds could have a mechanism of action based on RNA inhibition, we used NMR spectroscopy experiments to assess binding to a collection of six conserved structural elements of the SCoV2 RNA genome. The sequences included hairpins SL2 and SL3 of the 5’ untranslated region (UTR) (identified as 5_SL2+3), the programmed ribosomal frameshift full-length region (PRF) located between open reading frames ORF1a and ORF1b, a smaller PRF subdomain (ATTL), a segment of open reading frame 7b (ORF7b), and the SL1 and SL2 stem–loops (3_SL1+2) and S2m hairpin (3_S2m) of the 3’UTR (Figure 3 and Figure S3). The 5_SL2 hairpin is the most conserved structure in the 5’UTR, and the adjacent 5_SL3 stem–loop contains the transcription regulatory sequence (TRS) essential for subgenomic RNA (sgRNA) synthesis by discontinuous transcription [12]. The PRF element allows translation of the polymerase and other viral proteins coded by ORF1b by triggering ribosomal slippage by one nucleotide (nt) in the 5’ (−1) direction. While the 3’-half of the PRF can form a three-stem pseudoknot that stimulates frameshifting, the 5’-half (represented by sequence ATTL) contains the slippery site and an attenuator hairpin that reduces ORF1b translation [13]. Sequencing data [3,14] indicate that the selected ORF7b segment is likely involved in distal RNA-RNA interactions and transcriptional template switching in SCoV2. Stem–loops 3_SL1 and 3_SL2 of the 3’UTR are conserved in betacoronaviruses and have been suggested to form a pseudoknot that presumably functions as a molecular switch involved in negative-strand RNA synthesis [15]. The stem–loop II motif hairpin (3_S2m) is conserved in the Coronaviridae, Astroviridae, Caliciviridae, and Picornaviridae families of positive-sense single-stranded RNA viruses and has been proposed to be involved in host protein synthesis hijacking, RNA recombination events or cellular miRNA binding [16]. The secondary structure of all of these RNA elements has been determined in previous studies based on SHAPE profiling and NMR spectroscopy [4,17,18].

#### 2.4.2. Ligand-Based NMR Experiments

**Figure 4:**
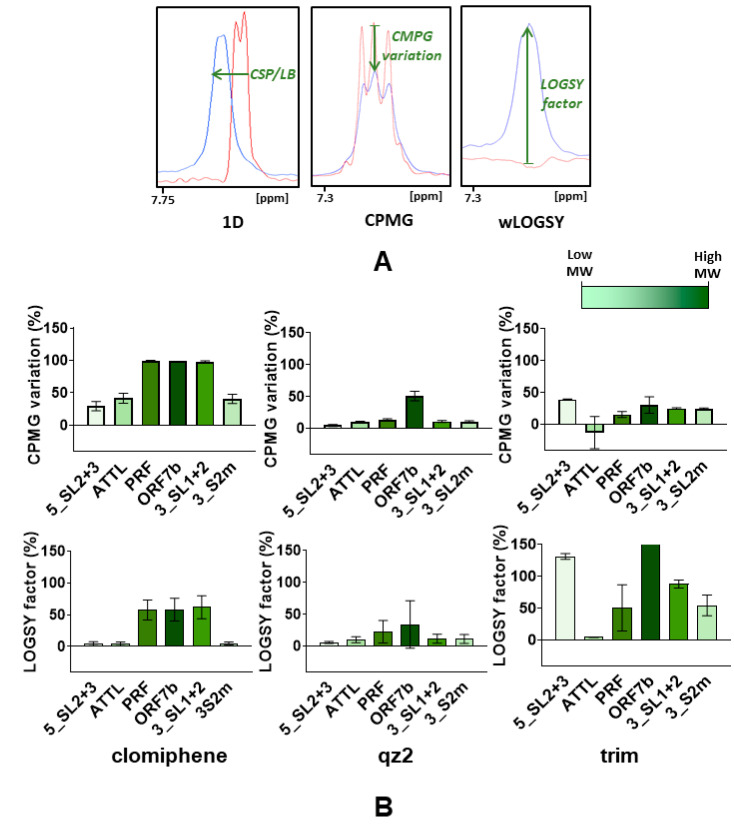
Interaction of clomiphene citrate, qz2 and trim with SCoV2 RNA elements analyzed by ligand-based NMR spectroscopy experiments. (**A**) Representative ^1^H one-dimensional (1D) CPMG, and wLOGSY spectra of trim in the absence (red) and presence (blue) of 5_SL2+3. RNA binding was assessed by quantifying chemical shift perturbation (CSP) or line broadening (LB) in 1D spectra, peak area variations in CPMG spectra, and LOGSY factors in wLOGSY spectra. (**B**) Quantification of CPMG and wLOGSY perturbations of aromatic protons as a function of compound and RNA element. RNA elements are ordered according to their relative locations in the virus genome. For RNA targets of similar size, increased binding translates into greater variations of CPMG areas and greater LOGSY factors. Note that both CPMG and LOGSY perturbations intensify with RNA target size. To account for this effect, the CPMG and LOGSY bars are colored according to the size of each RNA element as depicted in the image. The error bars represent the standard deviation of the average perturbation detected for the aromatic protons of each ligand. Conditions for (**A**,**B**): 300 μM compound; 3 μM RNA (1:100 molar ratio; clomiphene) or 6 μM RNA (1:50 molar ratio; trim and qz2); 150 mM KCl and 3 mM MgCl_2_; 100 ms CPMG delay; 27 °C.

We first used ligand-based ^1^H NMR experiments to analyze the interaction of the selected compounds with each of the six RNA elements specified above, including one-dimensional, Carr–Purcell–Meiboom–Gill (CPMG) and waterLOGSY (wLOGSY) experiments. These assays detect changes in the ^1^H signals of the ligand upon RNA association (Figure 4A), and have been successfully used to evaluate small-molecule binding to RNA motifs [19], including elements of the SCoV2 genome [20].

We evaluated RNA binding preferences by quantifying the one-dimensional (CSP and LB), CPMG and wLOGSY perturbations induced by each RNA element (Figure 4, Figures S4 and S5 and Table S3). Considering the relative molecular weights of the RNA molecules, the quantitative results suggested that trim bound with enhanced affinity to the 5_SL2+3 and 3_S2m molecules relative to other elements of similar or greater size, such as ATTL or PRF. Clomiphene citrate bound significantly to all of the RNA sequences examined, with the perturbations being principally modulated by the molecular weight of the RNA present in the mixture, which influenced the T_2_-relaxation changes observed in the ligand signals [19]. In contrast, the one-dimensional (CSP and LB), CPMG and wLOGSY quantifications indicated that qz2 bound to the ORF7b element only (Figure 4B and Figure S5 and Table S3).

The perturbation data also provided relevant information about the ligand components exhibiting enhanced RNA interactions. For example, of the two stereoisomers present in clomiphene citrate, the cis isomer bound RNA with stronger affinity than the trans form (Figure S6). In qz2, the strongest perturbation detected in one-dimensional (CSP and LB), CPMG and wLOGSY experiments with ORF7b involved the aromatic protons of the ligand (Figure 4B and Figure S5 and Table S3).

#### 2.4.3. RNA-Based NMR Experiments

**Figure 5:**
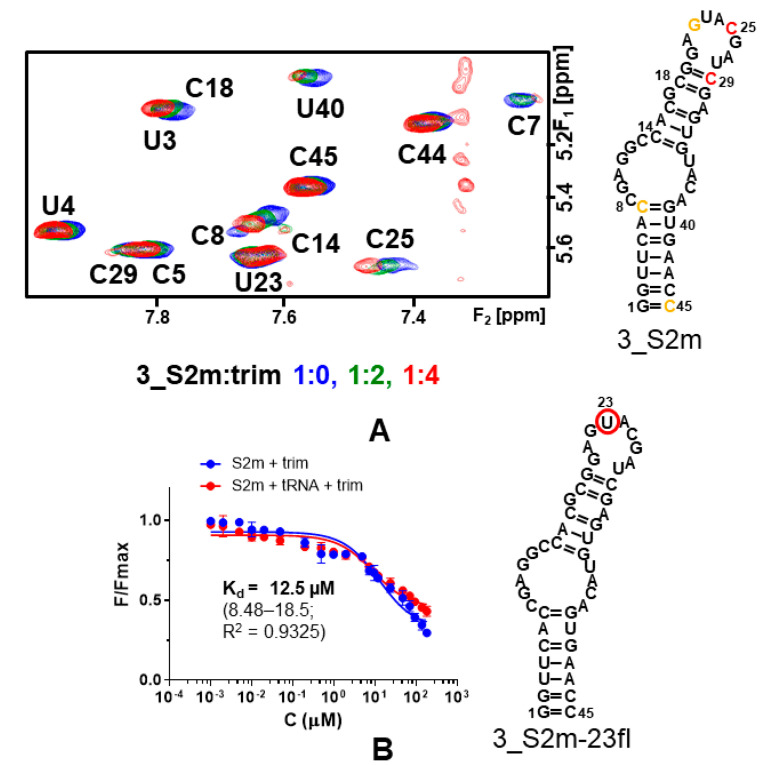
Recognition of the SCoV2 3’UTR S2m stem–loop by trim. (**A**) Interaction between 3_S2m and trim monitored by NMR spectroscopy. The pyrimidine H5-H6 region of the TOCSY spectrum (60 ms mixing time, 25 °C) of 3_S2m (blue) is superposed on the spectra of complexes with increasing RNA–ligand molar ratios, color-coded as indicated in the graph. On the right, the location of the ligand binding sites in the 3_S2m hairpin are indicated by highlighting the nt whose protons underwent broadening or chemical shift variations greater than two (orange) and three (red) standard deviations from the mean perturbation (0.028 and 0.042 ppm) upon the addition of four equivalents of ligand. To map the binding sites, we monitored the chemical shift variations of 63 aromatic, H1’, and H2’ 3_S2m RNA resonances with TOCSY and NOESY experiments. Conditions: 120 μM 3_S2m, 50 mM KCl, 25 °C. (**B**) Interaction between 3_S2m-23fl and trim monitored by fluorescence intensity. The experiments were carried out in the absence (blue) and presence (red) of a 100-fold molar excess of unlabeled competitor tRNA. The error bars represent standard deviations of three independent experiments. Conditions: 100 nM 3_S2m-23fl, 150 mM NaCl, 25 °C.

To confirm whether there was preferred binding to conserved viral RNA elements, we next used RNA-based ^1^H NMR experiments to examine the association of trim, clomiphene, and qz2 to the 5_SL2+3 and 3_S2m RNA elements, together with the attenuator hairpin of the PRF (ATTH; Figure 3B), which was used as a control. In line with the results of ligand-based experiments, the strongest RNA chemical shift perturbations were detected for the interaction between trim and 3_S2m. This compound induced small broadening or chemical shift variations that particularly affected the resonances of nt G22, C25, and C29 located in the apical loop of the hairpin (Figure 5A). Both clomiphene and qz2 induced smaller perturbations in 3_S2m. We detected weaker TOCSY perturbations in the 5_SL2+3 element relative to 3_S2m, with again trim inducing larger variations than clomiphene or qz2. No significant perturbations were detected in the ATTH element in the presence of any of the three compounds (Figure S7).

#### 2.4.4. Fluorescence Binding Assays

Since the NMR experiments suggested enhanced binding of trim to the apical loop of 3_S2m, we next quantified 3_S2m binding affinity by examining with fluorescence intensity assays the interaction of each compound with a 3_S2m hairpin containing a fluorescein probe at unpaired apical loop residue U23 (identified as 3_S2m-23fl; Figure 5B).

The results indicated that trim bound to 3_SL2m-23fl with a dissociation constant (K_d_) of 12.5 μM. The 3_S2m K_d_ value doubled for clomiphene citrate, 33.2 μM, whereas qz2 exhibited lower affinity for this RNA element (Figure 5B and Figure S8 and Table S4). The specificity of the trim and clomiphene interactions with 3_S2m was assessed by repeating the experiments in the presence of a 100-fold molar excess of tRNA. As shown in Figure 5B and Figure S8, we did not detect significant changes in the fluorescence curves in the presence of the tRNA competitor. In general, the 3_S2m affinities determined by the fluorescence binding assays followed the trend detected by the NMR experiments.

## 3. Discussion

At the time of writing, all authorized antiviral treatments for COVID-19 are based on direct-acting agents. These include the nucleoside analogs remdesivir and molnupiravir, targeting the viral RNA polymerase; nirmatrelvir, an inhibitor of the main SCoV2 protease; and several monoclonal antibodies blocking the spike protein of the virus. Additional protease and polymerase inhibitors, as well as several molecules targeting host factors, are also under advanced stages of clinical development [1,21,22,23,24]. However, the limitations of the currently approved treatments, the appearance of new SCoV2 variants, and the possibility of further pandemic outbreaks caused by other coronaviruses make the discovery of antiviral molecules with alternative mechanisms a necessity. Among the various possibilities for direct action, the functional structures formed by the SCoV2 RNA genome represent challenging targets that are starting to be interrogated [20,25,26,27,28,29].

In the context of the ongoing COVID-19 pandemic, we applied a phenotypic approach to identify SCoV2 inhibitors with RNA-binding properties. With this aim, a collection of 32 previously characterized HIV and HCV RNA-binding compounds was examined with a dose–response anti-SCoV2 immunofluorescence assay in Vero E6 cells. The compound library included 29 binders of the HIV-1 RRE, most of which inhibited the RRE-Rev complex and had substantial antiretroviral activity [5,6,7,8,9], and 3 ligands of the HCV IRES [10] (Table S1). The hit rate turned out to be exceptionally high, since 44% of the compounds had anti-SCoV2 EC_50_ values below 10 μM (Table S1). After a second evaluation in human 293T-ACE2 cells, eight compounds (25% of the initial set) exhibited an EC_50_ below 10 μM in both cell types and a selectivity index greater than 5 in at least one cellular type. These hit molecules included three compounds in current clinical use—clomiphene citrate, homochlorcyclizine, and triparanol—and five drug-like molecules—1d, 2a, trim, trityl-piperazine, and qz2 (Figure S2 and Table S2). Clomiphene citrate, homochlorcyclizine, and the 2-thienyl quinoline and trifluoropyridyl-piperazine compounds 1d and 2a were identified in experimental screens based on monitoring HIV-1 RRE-Rev inhibition [5,6], whereas trim, trityl-piperazine, and triparanol were detected in subsequent virtual searches based on a pharmacophore defined by clomiphene, homochlorcyclizine, and cyproheptadine [5,7]. All of these compounds bind RRE RNA subdomain IIB and, with the exception of homochlorcyclizine (with unknown antiretroviral activity), have anti-HIV EC_50_ values between 2 and 20 μM [5,6,7]. The antiretroviral mechanism of clomiphene citrate, in particular, was shown to be based on inhibition of LTR-dependent transcription and Rev function [5]. The piperidinyl-quinazolinone qz2, on the other hand, was identified as a ligand of the HCV IRES subdomain IIa [10]. These eight molecules represent valuable SCoV2 inhibitors from which to start chemical optimization. Indeed, previous studies have reported SCoV2 inhibitory activity for the FDA-approved compounds clomiphene citrate [30,31,32,33] and triparanol [31,32,33].

Based on anti-SCoV2 activities and selectivity indexes, we selected three compounds for further analyses: clomiphene citrate, trim, and qz2. These molecules exhibited SCoV2 antiviral EC_50_ values between 0.120 and 7.52 μM, depending on cell type (Figure 2A and Table S2), and reduced viral titers in infected cells at non-toxic concentrations (Figure 2B). Clomiphene was studied in Vero E6 and 293T-ACE2 cells as a stereoisomer mixture (the FDA-approved citrate salt), as well as in the pure cis and trans configurations. We found little difference in EC_50_ and SI values among the three forms (Table S2), so further analyses involved clomiphene citrate only because of its repurposing potential. 

In time-of-addition assays, the inhibition patterns detected for clomiphene citrate, trim, and qz2 were approximately similar to that observed for the viral RNA polymerase inhibitor remdesivir (Figure 2C). These patterns excluded action on the entry or egress stages of the virus replication cycle, indicating interference with a target, or set of targets, involved in the virus replication stage. To explore whether the selected compounds were able to bind SCoV2 RNA, we used ligand-based NMR spectroscopy experiments as a first tool to assess the interactions of these molecules with a collection of six conserved SCoV2 RNA domains, which comprised five functional stem–loops from the 5’- and 3’-UTR of the viral genome, two PRF sequences, and a segment of ORF7b likely involved in distal RNA-RNA interactions (Figure 3). The results indicated SCoV2 RNA association in all cases but were quite different depending on the molecule studied. Clomiphene citrate bound significantly to all six RNA elements with limited binding specificity, since the perturbations detected in the ligand signals were principally modulated by the molecular weight of the RNA target. For trim, the experiment suggested increased binding to the 3_S2m and 5_SL2+3 stem–loops located in the untranslated regions. qz2, on the other hand, bound selectively to the ORF7b segment (Figure 4 and Figure S5 and Table S3). Subsequent RNA-based NMR experiments analyzing the interactions of the compounds with 3_S2m and 5_SL2+3 revealed that trim induced greater RNA chemical shift perturbations relative to clomiphene and qz2 in these two RNA elements. Although the changes were small, the most significant ones were observed in nt located in the apical loop of 3_S2m (Figure 5A and Figure S7).

This 3’UTR stem–loop is highly conserved in coronaviruses and has been proposed to participate in recombination and cellular miRNA binding processes [16]. Fluorescence binding assays using a fluorophore-labeled S2m hairpin indicated that trim bound to the 3_S2m apical loop with a K_d_ of 12.5 μM, lower than the values measured for clomiphene (33.2 μM) or qz2 (>50 μM) in good agreement with the NMR analyses (Figure 5B and Figure S8 and Table S4). Docking calculations utilizing a 3_S2m RNA structural model [34] suggested that recognition of the 3_S2m loop by trim was mediated by a good steric fit of the three non-coplanar benzene rings in the major groove, a hydrogen-bonding contact to the floor of the groove involving the imidazole ring, and an electrostatic interaction between the amino-alkyl group and the RNA backbone. Elements of this binding mode are likely shared by four other molecules of the best-eight compound set, clomiphene citrate, trityl-piperazine, triparanol, and homochlorcyclizine, similarly composed of 2–3 non-coplanar benzene rings and a positively-charged amino group (Figure S2).

The K_d_ obtained for the interaction between trim and 3_S2m, 12.5 μM, is slightly above the antiviral EC_50_ of this compound in Vero E6 cells (3.5 μM) but well above the EC_50_ in human 293T-ACE2 cells (0.33 μM). This means that association with the 3’UTR S2m stem–loop is not sufficient to explain the antiviral mechanism of this molecule. We have explored a limited fraction of the structural RNA elements present in the SCoV2 genome, but while it is possible that trim binds with enhanced affinity to other viral elements, we cannot presently discard other antiviral mechanisms. In this regard, the association of this molecule to the 5_SL2+3 double hairpin, essential for sgRNA synthesis [12], or of qz2 to the ORF7b segment, merits further research.

The SCoV2 inhibitory action of clomiphene citrate has been previously proposed to be related to sigma receptor association [33] or to an unspecific mechanism based on drug-induced phospholipidosis [32]. We note in this latter respect that cationic amphiphilicity, the physicochemical property reported to drive phospholipidosis [32], likely drives RNA binding as well so that it is possible that viral RNA binding contributes to the observed antiviral effect of clomiphene and other FDA-approved drugs, even for unspecific compounds. In this regard, clomiphene is capable of inhibiting the replication of HIV-1, which does not associate with membranes and should not be affected by phospholipidosis. The anti-HIV EC_50_ of this compound, 4.4 μM, was close to its HIV-1 RNA-binding K_d_ value (12.4 μM for RRE subdomain IIB), and RT-PCR assays supported an antiviral mechanism based on inhibition of Rev function in addition to transcriptional blockage [5].

Chemical optimization of the trityl-imidazole hit is currently in progress in our laboratories, with the aim of improving antiviral activity and selectivity index, as well as pharmacokinetic properties. In this regard, in vivo antiviral experiments will be important to validate the potential of these compounds as antiviral agents.

## 4. Materials and Methods

### 4.1. Compounds

The source of the 32 HIV-1 and HCV RNA-binding compounds is specified in Table S1. The library contained 23 commercial molecules obtained from Sigma-Aldrich (St. Louis, USA), Prestwick Chemicals (Illkirch-Graffenstaden, France), Santa Cruz Biotechnology (Dallas, TX, USA), AKos GmbH (Stuttgart, Germany), MyriaScreen Diversity Collection of Sigma-Aldrich (St. Louis, MO, USA), Toronto Research Chemicals (Toronto, ON, Canada), BOC Sciences (Shirley, NY, USA), Princeton Biomolecular Research (Princeton, NJ, USA), and SIA Enamine (Riga, Latvia) ([5,6,7,10]; Table S1). The remaining compounds were 9 terphenyl mimics of the HIV-1 protein Rev, synthesized as described previously [8,9]. Remdesivir, used as a reference in the antiviral experiments, was obtained from Medkoo Biosciences (Morrisville, NC, USA). All compounds were dissolved in DMSO (Sigma-Aldrich, St. Louis, MO, USA) at a concentration of 5 mM, except for the terphenyl compounds, which were dissolved in H_2_O at the same concentration. Compounds aimed for NMR analyses were dissolved in DMSO-d6 (Deutero GMBH, Kastellaun, Germany) at 5 mM concentration.

### 4.2. Evaluation of Anti-SCoV2 Activity and Cellular Toxicity

Two thousand Vero E6 or 293T-ACE2 cells were seeded into 96-well plates in cell growth media (DMEM 10% FBS) and incubated for one day at 37 °C and 5% CO_2_. Two hours before infection with SARS-CoV-2 USA-WA1/2020, the medium was replaced with 100 µL of viral growth medium (DMEM 2% FBS) containing the indicated compounds at a concentration fifty percent greater than those indicated, together with a DMSO control. Experiments were then transferred into the Biosafety Level 3 facility, and 100 PFU (MOI = 0.025) for VeroE6 infections and 1000 PFU (MOI = 0.25) for 293T-ACE2 infections were added in 50 µL of viral growth media, bringing the final compound concentrations to those indicated. Plates were incubated for two days at 37 °C. Supernatants were then removed, and cells were fixed with 4% formaldehyde for one day prior to being removed from the Biosafety Level 3 facility. The cells were then immunostained for the viral N protein with an in-house mAb 1C7 provided by Dr. Thomas Moran (Thomas.Moran@mssm.edu), with a DAPI counterstain [35]. Infected cells (488 nM) and total cells (DAPI) were quantified using a Celigo imaging cytometer (Nexcelcom Bioscience, Lawrence, KS, USA). Infection was measured by quantification of cells expressing viral N protein (fluorescence accumulation). Percent infection was quantified as ((Infected cells/Total cells) − Background) ∗ 100, and the DMSO control was then set to 100% infection for analyses. The EC_50_ and EC_90_ for each experiment were determined using Prism software (GraphPad Software, San Diego, CA, USA). Cytotoxicity was also measured using the MTT assay (Roche, Basel, Switzerland), according to the manufacturer’s instructions. Cytotoxicity experiments were performed in uninfected cells with identical compound dilutions and concurrently with the antiviral assay. Infectious SCoV2 in supernatants was measured using a TCID_50_ assay. Briefly, infectious supernatants were collected at 48 h post-infection in the absence and presence of two compound concentrations and frozen at −80 °C. Infectious titers were quantified by limiting dilution titration using Vero E6 cells as previously described [36]. All assays were performed in biologically independent triplicates.

### 4.3. Time-of-Addition Assays 

Two thousand 293T-ACE2 cells were seeded in 96-well plates. One day later, cells were infected with SCoV2 USA-WA1/2020 at an MOI of 2. DMSO vehicle or indicated compounds were added at different time points relative to infection, according to the timeline and concentrations indicated in Figure 2C. Cells were fixed at 8 h post-infection and subjected to an immunofluorescence assay targeting SCoV2 N protein to quantify the percentage of infected cells, as described in Section 4.2. 

### 4.4. Preparation of SCoV2 RNA Samples for NMR Spectroscopy and Fluorescence Experiments

The following sequences of the SCoV2 RNA genome were prepared by T7-polymerase in vitro transcription for NMR analyses: SL2 and SL3 hairpins of the 5’UTR (nt 45-75; 5_SL2+3, 32 nt), programmed ribosomal frameshift (PRF) region (nt 13420-13542; PRF, 124 nt), attenuator and slippage region of the PRF region (nt 13425-13476; ATTL, 56 nt), attenuator hairpin of the PRF region (nt 13432-13455; ATTH, 26 nt), ORF7b segment (nt 27694-27867; ORF7b, 176 nt), SL1 and SL2 hairpins of the 3’UTR (nt 29543-29657; 3_SL1+2, 115 nt), and S2m hairpin of the 3’UTR (nt 29728-29768; 3_S2m, 45 nt) (Figure 3B and Figure S3). The DNA templates used in the transcription reactions were generated by PCR (PRF, ORF7b, and 3_SL1+2 RNA sequences) or obtained commercially from Integrated DNA Technologies (Coralville, IA, USA) (5_SL2+3, ATTL, ATTH, and 3_S2m). All RNA constructs were purified on denaturing polyacrylamide gels containing 8 M urea, followed by electroelution, ethanol precipitation (twice), and desalting with Sephadex G-25 cartridges (Cytiva, Marlborough, MA, USA). Prior to NMR experiments, all samples were transferred by diafiltration into an aqueous solution containing 25 mM potassium phosphate (pH 6.2), 50 or 150 mM KCl, and 0 or 3 mM MgCl_2_.

The 3_S2m-23fl RNA oligonucleotide used in fluorescence intensity experiments was purchased HPLC-purified from Integrated DNA Technologies (Coralville, IA, USA). This oligonucleotide contained a fluorescein probe linked to the apical loop nt U23 and was identical in sequence to the unlabeled 3_S2m molecule used in NMR experiments (Figure 5). The mixture of tRNAs from *E. coli* MRE 600 (identified as tRNA^mix^) used to evaluate specificity was obtained from Roche (Basel, Switzerland).

### 4.5. NMR Spectroscopy

NMR spectra were acquired in Avance III 500 MHz and cryoprobe-equipped Avance II 600 MHz spectrometers (Bruker, Billerica, MA, USA), and analyzed using Topspin, version 3.6.1 (Bruker, Billerica, MA, USA) and NMRFAM-Sparky, version 1.414 (University of Wisconsin, Madison, WI, USA) [37] software packages. 

#### 4.5.1. Ligand-Based Experiments

One-dimensional, relaxation-edited Carr–Purcell–Meiboom–Gill (CPMG) and water-ligand observed by gradient spectroscopy (wLOGSY) ^1^H experiments [19] were used to evaluate the interaction of clomiphene citrate, trim, and qz2 with six SCoV2 RNA sequences at 27 °C. Each ligand (at a concentration of 300 μM) was mixed with a smaller quantity of RNA in an aqueous solution containing 25 mM potassium phosphate (pH 6.2), 150 mM KCl, 3 mM MgCl_2_, and 10% D_2_O. RNA–ligand molar ratios of 1:50 and 1:100 were explored (with 6 and 3 μM RNA, respectively). Clomiphene citrate was studied at a 1:100 ratio to avoid precipitation observed in the 1:50 condition. In all cases, either N-methyl-valine (Sigma-Aldrich, St. Louis, MO, USA) [19] or the citrate ion of clomiphene citrate was used as internal negative-binding controls. The water signal was suppressed from one-dimensional experiments with a watergate sequence. The CPMG experiments were acquired with 100, 200, 300, and 400 ms relaxation delays before acquisition, and water suppression was achieved by presaturation [19]. wLOGSY experiments employed a selective Gaussian 180° pulse at the water frequency and an NOE mixing time of 1.5 s and suppressed the water signal by excitation sculpting [19,38]. All spectra were acquired in the absence and presence of RNA.

All ligand signals were individually analyzed. For clomiphene citrate, the signals corresponding to cis and trans forms were identified by comparing the spectrum of clomiphene citrate with those of the individual stereoisomers. In CPMG experiments, the binding of the compounds to the different RNA sequences was quantified as 100 minus the percentage of ligand signal area in the +RNA CPMG spectrum relative to the corresponding signal area in the -RNA CPMG spectrum. In wLOGSY experiments, binding was quantified with the LOGSY factor, or % of ligand signal in the difference spectrum between the +RNA and –RNA wLOGSY spectra, relative to the corresponding signal in the one-dimensional -RNA reference spectrum [39]. In one-dimensional experiments, line broadening (LB) was quantified as explained for CPMG spectra. 

#### 4.5.2. RNA-Based Experiments

The interactions of trim, clomiphene citrate, and qz2 with 3_S2m, 5_SL2+3, and ATTH RNA molecules were monitored with one- and two-dimensional (TOCSY; 60 ms mixing time) experiments at increasing RNA–ligand molar ratios of 1:0, 1:1, 1:2 and 1:4. The RNA samples were previously diafiltrated into an aqueous solution containing 25 mM potassium phosphate (pH 6.2), 50 mM KCl and 0.1 mM EDTA, and the titration experiments were carried out at 27 °C using 50 μM RNA samples in 100% D_2_O. The 3_S2m titrations were duplicated at 25 °C using 120 μM RNA samples and additionally acquiring NOESY spectra (250 ms mixing time) at 1:0 and 1:4 ratios. For this system, the chemical shift perturbation analyses included 63 H1’, H2’, and aromatic 3_S2m resonances. The isolated 3_S2m hairpin was studied at two different temperatures (25 and 35 °C) using 120 and 500 μM RNA samples and similar TOCSY and NOESY (120 and 250 ms mixing times) experiments. The assignment of 3_S2m non-exchangeable ^1^H resonances was based on the analysis of these spectra, as well as on previously published assignments of 3_S2m exchangeable protons [4]. A similar procedure was conducted for assigning non-exchangeable protons of the 5_SL2+3 and ATTH RNA elements. For 5_SL2+3, we also used as support published assignments of the 5_SL2 hairpin [40]. The relaxation delay for NOESY and TOCSY acquisition was 2 and 1.5 s, respectively.

### 4.6. Fluorescence Binding Assays

These experiments measured the association of clomiphene citrate, trim, and qz2 with a 3_S2m-23fl RNA hairpin labeled with fluorescein at extrahelical loop nucleotide U23 (Figure 5B) and were carried out at 25 °C and under two ionic conditions in a Victor X5 plate reader (Perkin Elmer, Waltham, MA, USA), using excitation and emission wavelengths of 485 and 535 nm, respectively. Except for qz2 at concentrations above 50 μM, the compounds did not fluoresce in these conditions. 3_S2m-23fl (at 100 nM concentration) was snap-cooled in a buffer containing sodium phosphate pH 6.6, 0 or 150 mM NaCl, and 0.1 mM EDTA and incubated with increasing amounts of compound. The specificity of the RNA–ligand interactions was evaluated by repeating the assays in the presence of a 100-fold molar excess (10 μM) of tRNA^mix^. The equilibrium dissociation constants K_d_ were obtained by fitting the dose–response curves to a two-state binding model [41] with Prism software (GraphPad Software, San Diego, CA, USA). All fluorescence binding assays were carried out three times for each molecule and condition.

### 4.7. Molecular Modeling

A three-dimensional model of a 3_S2m-trim complex was built starting from a 3_S2m FARFAR2 model structure previously generated [34] using secondary structure information from NMR spectroscopy [4]. The potential energy of this structure was minimized with the ff99-OL3 force field of AMBER prior to docking trim with Gold, version 2020 software (CCDC, Cambridge, UK) [42]. The docking calculations were unrestrained, defined the ligand binding site with a 19 Å radius around apical loop nt A21, and employed the GoldScore fitness function.

## 5. Patents

A patent application, PCT/EP2022/053938, partially resulted from the work reported in this manuscript.

## 6. Conclusions

By screening a small library of HIV and HCV RNA-binding compounds with a dose–response live-SCoV2 assay, we have identified eight FDA-approved and drug-like molecules with significant antiviral activities and selectivity indexes. Three of the screening hits had substantial anti-SCoV2 activity and associated with functional SCoV2 RNA elements with different degrees of selectivity. While further studies will be needed to determine the antiviral mechanism of action of these compounds, these results open the door to the development of new anti-ScoV2 agents with a mode of action different from those used by typical direct-action agents. 

## Data Availability

Data are contained within the article and Supplementary Material.

## Supplementary Materials

The following supporting information can be downloaded at: https://www.mdpi.com/article/10.3390/ph15121448/s1, Figure S1: dose-response SCoV2 antiviral activity and toxicity curves of viral RNA-binding small-molecule compounds, determined in Vero E6 and h293T-ACE2 cells; Figure S2: chemical structure of 8 selected compounds identified by phenotypic screening; Figure S3: SCoV2 RNA sequences studied in this report; Figure S4: one-dimensional ^1^H NMR spectroscopy spectra of trim, qz2 and clomiphene citrate, indicating the signals used to quantify the perturbations detected in one-dimensional, CPMG and wLOGSY experiments in the presence of different SCoV2 RNA elements; Figure S5: interaction of clomiphene citrate, qz2 and trim with SCoV2 RNA elements analyzed by ligand-based NMR spectroscopy experiments; Figure S6: interaction of the cis and trans stereoisomers of clomiphene citrate with SCoV2 RNA elements analyzed by ligand-based NMR spectroscopy experiments; Figure S7: recognition of 3_S2m, 5_SL2+3 and ATTH by clomiphene and trim, monitored by RNA-based NMR spectroscopy experiments; Figure S8: 3_S2m recognition by trim, clomiphene citrate and qz2, studied with fluorescence intensity experiments at two ionic strength conditions, 0 and 150 mM NaCl; Table S1: antiviral activity, toxicity and previous characterization of the 32 viral RNA-binding small-molecule compounds analyzed by phenotypic screening; Table S2: antiviral activity and toxicity of the best compounds identified by screening a library of 32 viral RNA-binding compounds; Table S3: quantitative analysis of the perturbations detected in the ^1^H NMR spectra of clomiphene citrate, qz2 and trim as a function of the SCoV2 RNA element present in the mixture; Table S4: 3_S2m RNA interaction parameters of trim, clomiphene and qz2, measured by fluorescence intensity experiments.
