# Supplementary material for: SARS-CoV-2 Inhibitors Identified by Phenotypic Analysis of a Collection of Viral RNA-Binding Molecules
Source: Pharmaceuticals (Basel). 2022 Nov 22;15(12):1448. doi: 10.3390/ph15121448 (PMC9784969; doi:10.3390/ph15121448)
Supplement: Supplementary file 1 [file pharmaceuticals-15-01448-s001.zip › pharmaceuticals-1998476-sm211122-clean.pdf]

SARS-CoV-2 inhibitors identified by phenotypic analysis of a collection of viral RNA-binding molecules

Alvaro Simba-Lahuasi, Ángel Cantero-Camacho, Romel Rosales, Briana Lynn McGovern, M. Luis Rodríguez, Vicente Marchán, Kris M. White*, Adolfo García-Sastre and José Gallego*

SUPPLEMENTARY MATERIAL

Supplementary Figures

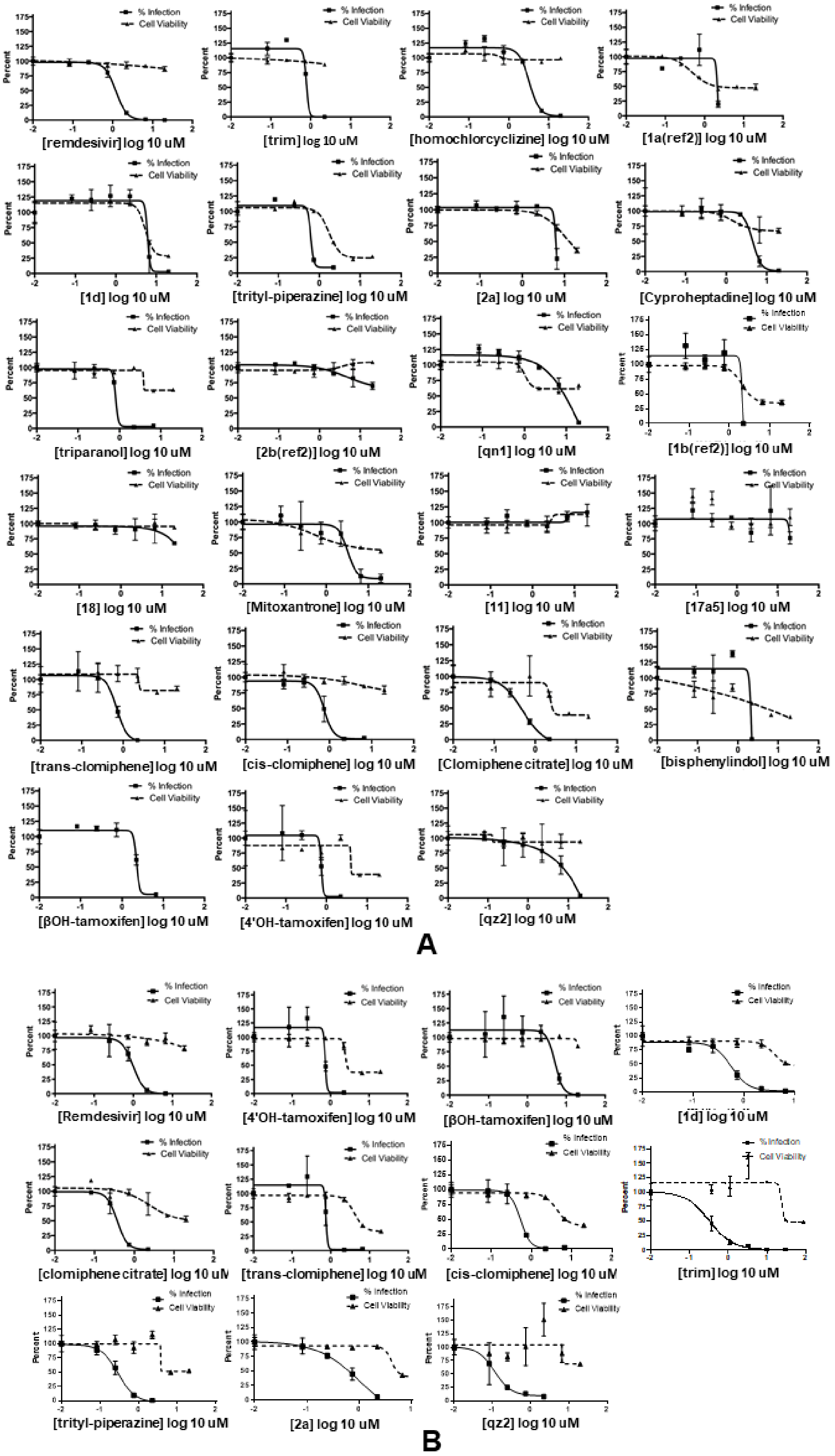

Figure S1. Dose-response SCoV2 antiviral activity and toxicity curves of viral RNA-binding small-molecule compounds, determined in Vero E6 (A) and h293T-ACE2 (B) cells.

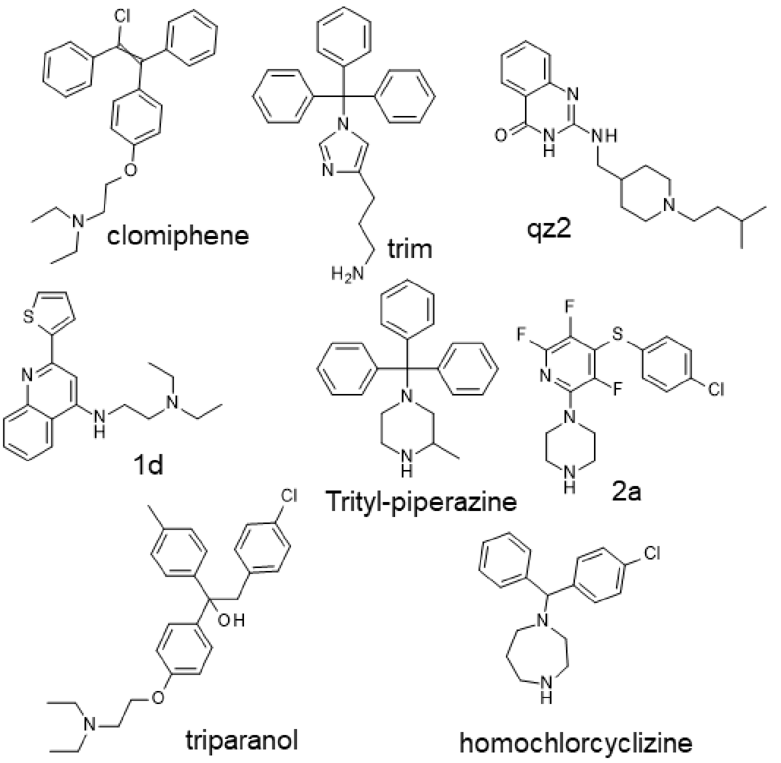

Figure S2. Chemical structure of 8 selected compounds identified by phenotypic screening. These molecules exhibited anti-SCoV2 $EC_{50} < 10$ μM in Vero E6 and h293T-ACE2 cells, and SI > 5 in at least one cell type, and represent 25% of the screening library. Clomiphene was studied in the cis and trans configurations as well as a stereoisomer mixture (FDA-approved citrate salt).

RNA	sequence
5_SL2+3	5' GGAUCUCUUGUAGAUCUGUUCUCUAAACGAAC 3'
PRF	5' GACUCCGCGAACCCAUGCUUCAGUCAGCUGAUGCACAAUCGUUUUUAAACGGGUUUGCG GUGUAAGUGCAGCCCGUCUUACACCGUGCGGCACAGGCACUAGUACUGAUGUCGUAUACAG GGCU 3'
ATTL	5' GGGCGAACCCAUGCUUCAGUCAGCUGAUGCACAAUCGUUUUUAAACGGGUUUGCCC 3'
ATTH	5' GGUGCUUCAGUCAGCUGAUGCACC 3'
ORF7b	5' GGUUUCUUAUUGUUGCGGCAAUAGUGUUUAUAACACUUUGCUUCACACUCAAAAGAAACC CAGAAUGAUUGAACUUUCAUUAAUUGACUUCUAUUUGUGCUUUUUAGCCUUUCUGCUAUUC CUUGUUUUAAUUAUGCUUAUUAUCUUUUGGUUCUCACUUGAACUGCAAGAUCAUA 3'
3_SL1+2	5' GACCACACAAGGCAGAUGGGCUAUAUAAACGUUUUCGCUUUUCCGUUUACGAUAUAUAGU CUACUCUUGUGCAGAAUGAAUUCUCGUAACUACAUAGCACAAGUAGAUGUAGUUA 3'
3_S2m	5' GGUUCACCGAGGCCACGCGGAGUACGAUCGAGUGUACAGUGAACC 3'

Figure S3. SCoV2 RNA sequences studied in this report. The blue-colored nucleotides indicate changes relative to the wild-type sequence, introduced to increase transcription yield.

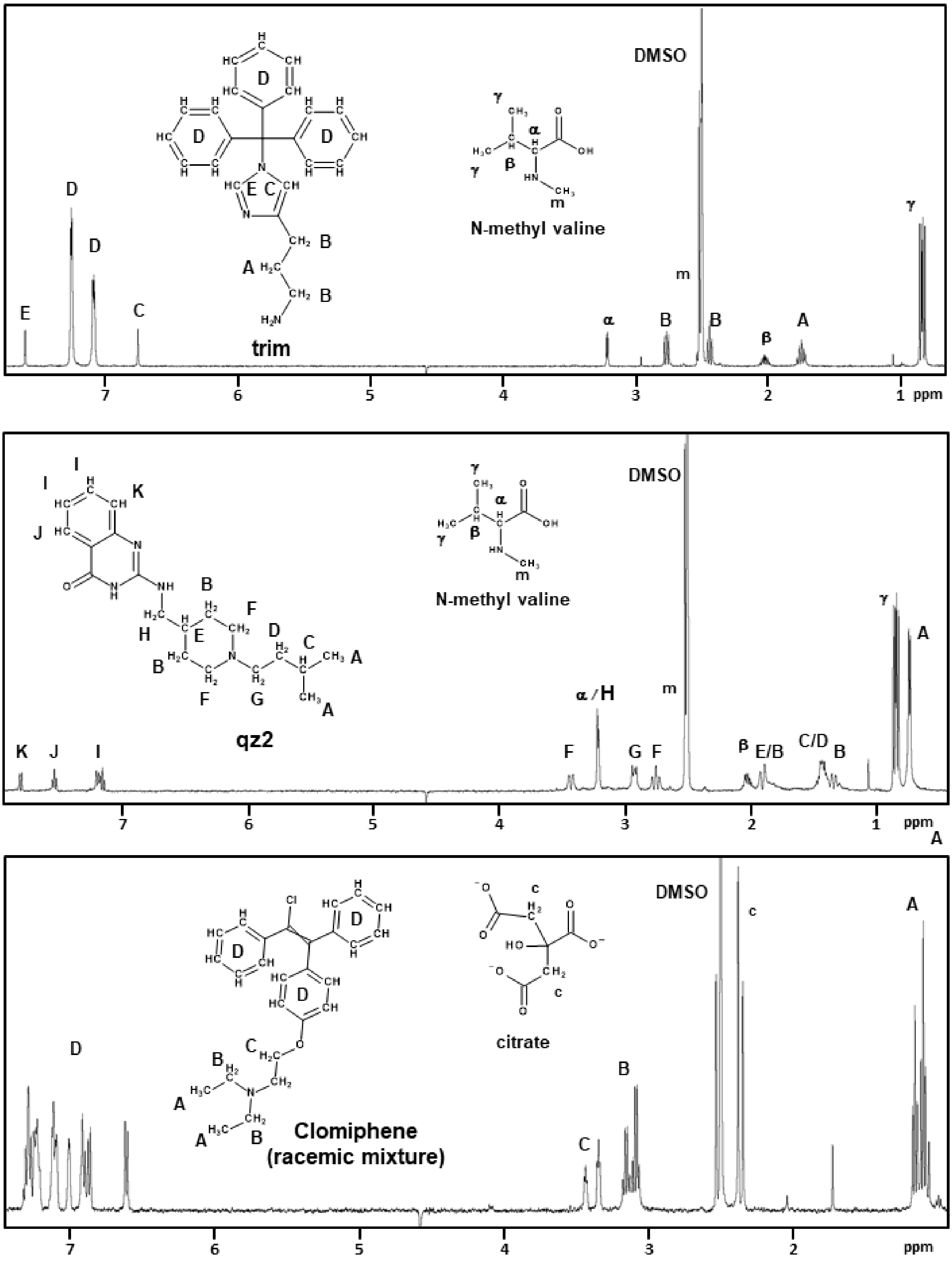

Figure S4. One-dimensional ^{1}H NMR spectroscopy spectra of trim, qz2 and clomiphene citrate, indicating the signals used to quantify the perturbations detected in one-dimensional, CPMG and wLOGSY experiments in the presence of different SCoV2 RNA elements. Conditions: 300 µM compound; 150 mM KCl and 3 mM $MgCl_2$, 27 ºC. The resonances belonging to N-methyl-valine or the citrate salt of clomiphene, used as internal negative-binding controls, are also assigned.

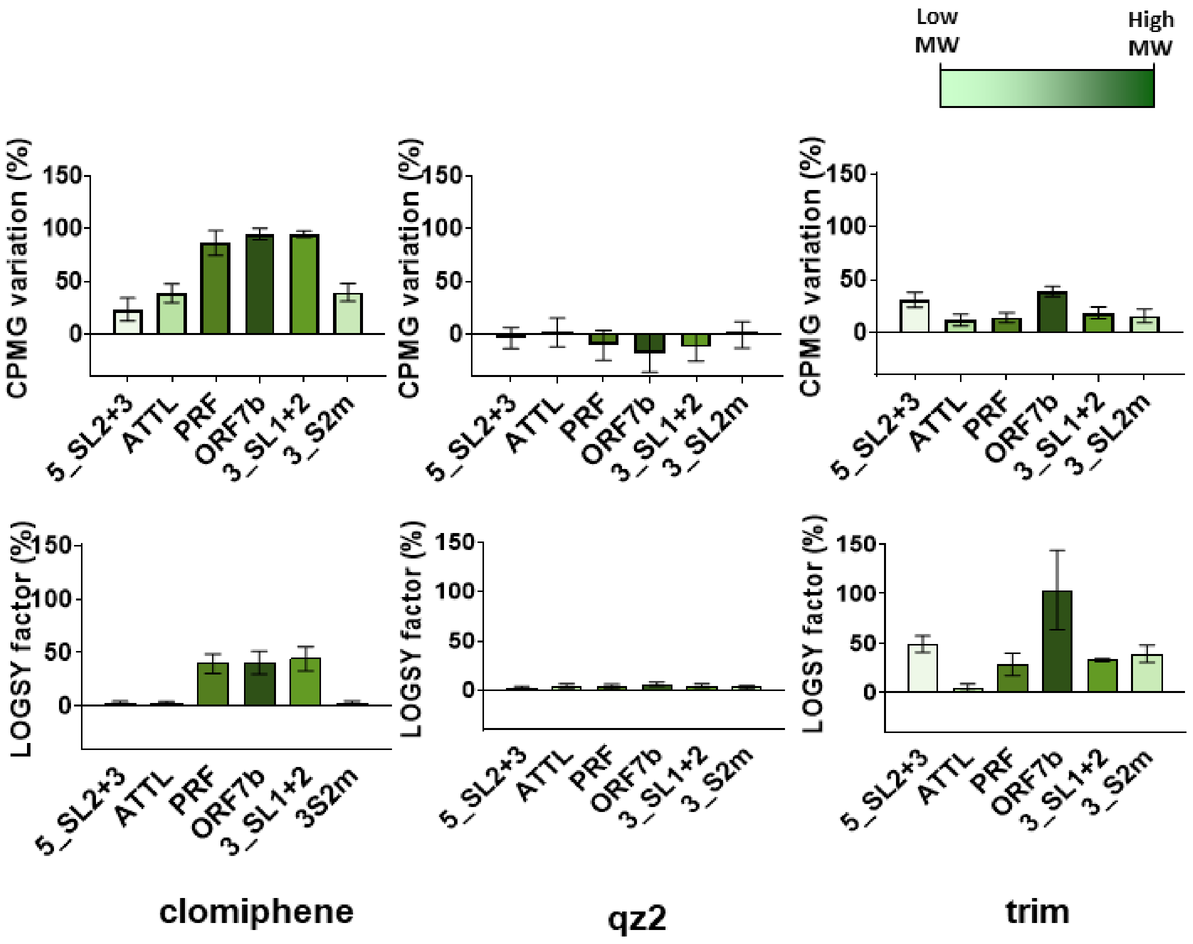

Figure S5. Interaction of clomiphene citrate, qz2 and trim with SCoV2 RNA elements analyzed by ligand-based NMR spectroscopy experiments. Quantification of CPMG and wLOGSY perturbations of aliphatic protons as a function of compound and RNA element. RNA elements are ordered according to their relative locations in the virus genome. For RNA targets of similar size, increased binding translates into greater variations of CPMG areas and greater LOGSY factors. Note that both CPMG and LOGSY perturbations intensify with RNA target size. To account for this effect, the CPMG and LOGSY bars are colored according to the size of each RNA element as depicted in the image. The error bars represent the standard deviation of the average perturbation detected for the aliphatic protons of each ligand. Conditions: 300 μM compound; 3 μM RNA (1:100 RNA:ligand molar ratio; clomiphene) or 6 μM RNA (1:50 molar ratio; trim and qz2); 150 mM KCl and 3 mM $MgCl_2$; 100 ms CPMG delay; 27 °C.

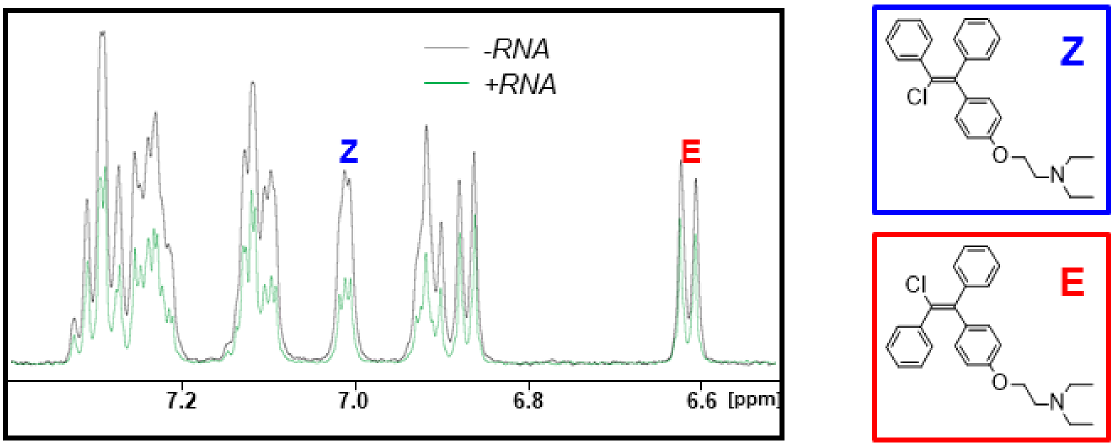

A

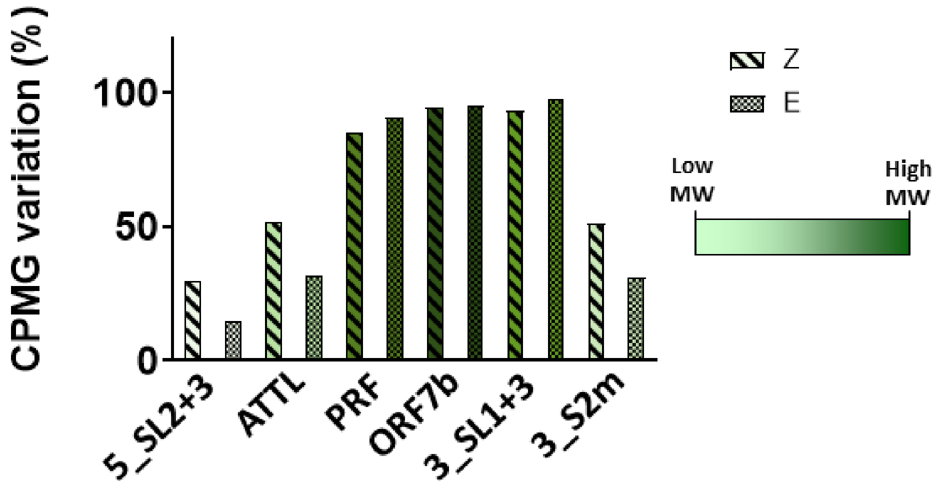

B

Figure S6. Interaction of the cis (Z) and trans (E) stereoisomers of clomiphene citrate with SCoV2 RNA elements analyzed by ligand-based NMR spectroscopy experiments. (A) Representative CPMG spectra of clomiphene citrate in the absence (black) and presence (green) of 3_SS2m. Two resolved aromatic peaks corresponding to the E and Z stereoisomers are perturbed differently. (B) Quantification of CPMG perturbations of E and Z resonances as a function of SCoV2 RNA element. RNA elements are ordered according to their relative locations in the virus genome. For RNA targets of similar size, increased binding translates into greater variations of CPMG areas. Note that CPMG perturbations intensify with RNA target size. To account for this effect, the bars are colored according to the size of each RNA element as depicted in the image. Two pairs of aromatic and aliphatic peaks corresponding to the E and Z stereoisomers were used for quantification. Conditions for (A) and (B): 3 µM RNA and 300 µM compound (1:100 molar ratio); 150 mM KCl and 3 mM $MgCl_2$; 100 ms CPMG delay; 27 ºC.

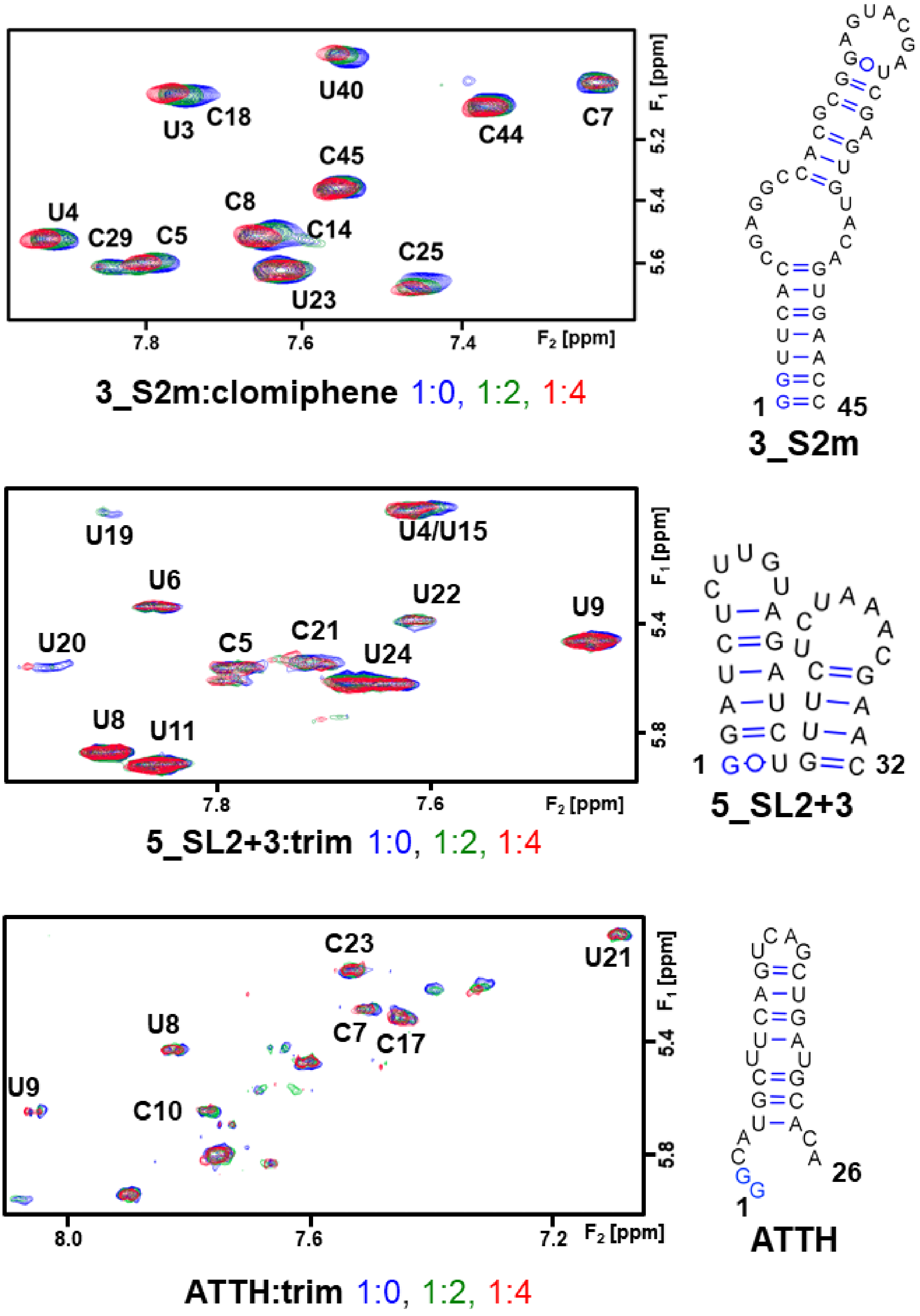

Figure S7. Recognition of 3_S2m, 5_SL2+3 and ATTH by clomiphene and trim, monitored by RNA-based NMR spectroscopy experiments. In all cases, the H5-H6 region of the TOCSY spectrum (60 ms mixing time) of each RNA element is superposed on the spectra of complexes with increasing RNA:ligand molar ratios, color-coded as indicated in the graphs. Conditions: 120 µM (3_S2m) or 50 µM (5_SL2+3, ATTH) RNA, 50 mM KCl, 25 ºC (3_S2m) or 27 ºC (5_SL2+3, ATTH).

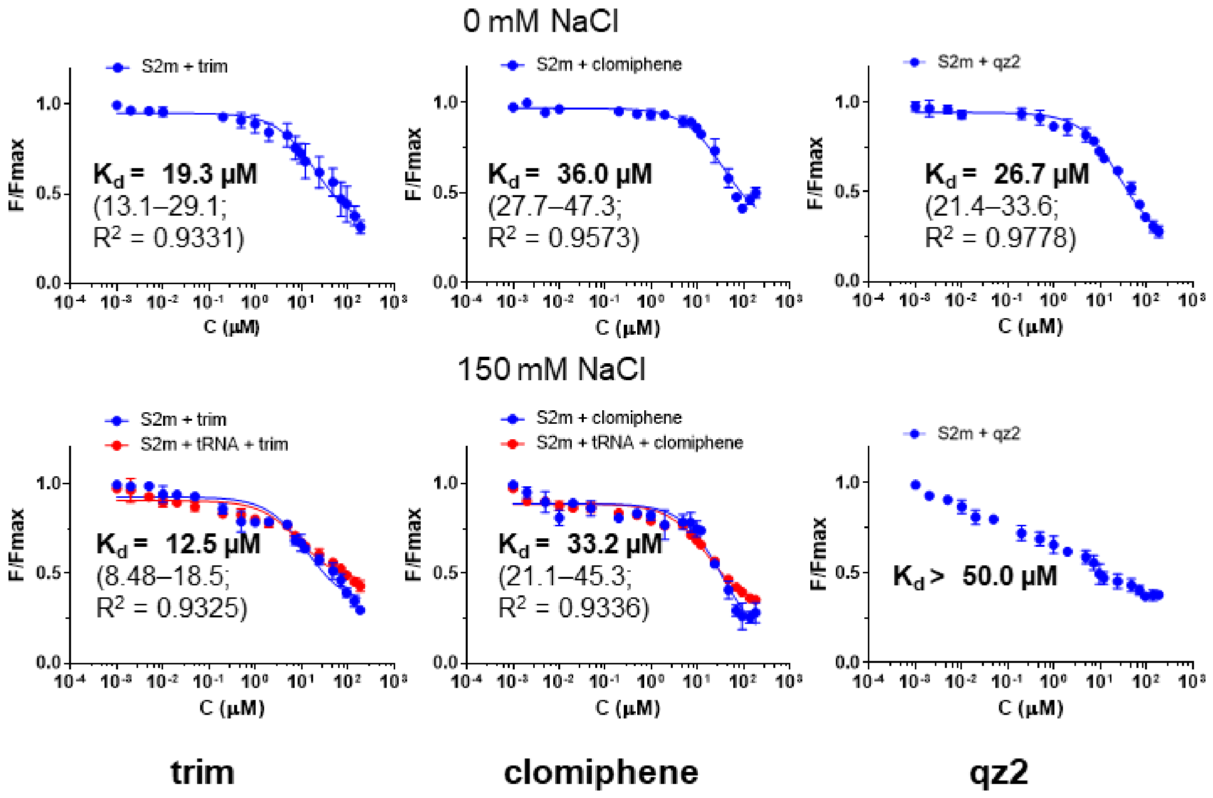

Figure S8. 3_S2m recognition by trim, clomiphene citrate and qz2, studied with fluorescence intensity experiments at two ionic strength conditions, 0 and 150 mM NaCl. No clear transition is observed for qz2 at higher ionic strength. For trim and clomiphene at 150 mM NaCl, the graphs show 3_S2m association curves obtained in the absence (blue circles) and presence (red circles) of a 100-fold molar excess of unlabeled competitor tRNA. The presence of tRNA did not have a significant impact on 3_S2m binding in either case. Conditions: pH 6.6, 0 or 150 mM NaCl, 25 ºC.

Supplementary Tables

Table S1. Antiviral activity, toxicity and previous characterization of the 32 viral RNA-binding small-molecule compounds analyzed by phenotypic screening. The table is supplied separately in excel format and provides, for each compound, SCoV2 inhibitory activity (EC_{50}), toxicity (CC_{50}) and selectivity index ($SI = CC_{50}/EC_{50}$) in Vero E6 and 293T-ACE2 cells, previous virus and RNA target information, smiles code and source. The SCoV2 inhibitory data of remdesivir, used as a reference, is also provided.

Table S2. Antiviral activity and toxicity of the best compounds identified by screening a library of 32 viral RNA-binding compounds. Anti-SCoV2 activity (EC_{50}), cellular toxicity (CC_{50}) and selectivity index (SI) values were measured in Vero E6 and human 293T-ACE2 cells. The 8 unique hits included in this set had an EC_{50} < 10 µM in both cell types and SI > 5 in at least one cellular type and represent 25% of the compound library.

Compound[a]	*EC_{50} Vero* (µM)	*CC_{50} Vero* (µM)	*SI Vero*	*EC_{50} 293T* (µM)	*CC_{50} 293T* (µM)	*SI 293T*	*Previous viral target*	*Refere nce*
clomiphene citrate[b]	0.449	2.62	5.84	0.371	>20	>53.9	HIV-1 RRE	[1]
trans-clomiphene[b]	0.760	5.58	7.34	0.210	47.1	224	HIV-1 RRE	[1]
cis-clomiphene[b]	0.590	6.86	11.6	0.520	6.87	13.2	HIV-1 RRE	[1]
trim	3.5	>89.8	>25.7	0.33	23.5	71.2	HIV-1 RRE	[2]
qz2	7.52	>20	>2.7	0.12	>6.6	>55	HCV IRES	[3]
trityl-piperazine	0.644	2..22	3.45	0.270	>3.67	>13.6	HIV-1 RRE	[2]
homochlorcyclizine	3.59	>20	>5.57	n/d	n/d	n/d	HIV-1 RRE	[1]
triparanol	0.811	>20	>24.7	n/d	n/d	n/d	HIV-1 RRE	[2]
2a	6.24	>20	>3.21	0.63	5.32	8.44	HIV-1 RRE	[4]
1d	6.64	6.86	1.10	0.520	7.50	14.4	HIV-1 RRE	[4]

[a]The EC_{50}, CC_{50} and SI values obtained for the control antiviral remdesivir were 0.428 µM, >20 µM and > 46.7 in Vero E6 cells, and 0.0104 µM, >2µM and >192 in h293T-ACE2 cells, respectively.
[b]Clomiphene was studied in the cis and trans forms as well as a stereoisomer mixture (FDA-approved citrate salt).

Table S3. Quantitative analysis of the perturbations detected in the ^{1}H NMR spectra of clomiphene citrate, qz2 and trim as a function of the SCoV2 RNA element present in the mixture. The table includes line broadening (LB), CPMG and wLOGSY perturbation values, averaged for the aromatic and aliphatic protons of the ligands (section A), or measured for the assigned peaks indicated in Figure S4 (section B). In the latter case chemical shift perturbation (CSP) values are also reported. Conditions: 300 µM compound; 3 µM RNA (1:100 RNA:ligand molar ratio; clomiphene) or 6 µM RNA (1:50 molar ratio; trim and qz2); 150 mM KCl and 3 mM $MgCl_2$; 100 ms CPMG delay; 27 ºC.

A

Aromatic	*Clomiphene citrate*			*qz2*			*trim*		
Sequence	*LB (%)*	*LOGSY factor (%)*	*CPMG variation (%)*	*LB (%)*	*LOGSY factor (%)*	*CPMG variation (%)*	*LB (%)*	*LOGSY factor (%)*	*CPMG variation (%)*
5_SL2+3	-1.34 ± 9.47	4.44 ±3.00	29.2 ±7.19	1.97 ± 7.15	5.80 ± 1.62	4.67 ± 1.53	15.9 ± 1.21	131.1 ± 4.61	39.0 ± 0.82
ATTL	15.9 ± 12.5	4.11 ±3.05	41.4 ±7.70	3.39 ± 8.56	9.98 ± 4.61	10.0 ± 1.00	9.31 ± 9.31	4.35 ± 0.32	-12.7 ± 25.2
PRF	81.7 ± 10.3	57.5 ±15.8	99.0 ±1.00	-0.27 ± 1.42	22.5 ± 17.6	13.0 ± 2.00	-22.5 ± 31.62	50.5 ± 36.2	15.5 ± 4.80
ORF7b	67.6 ± 6.22	58.0 ±18.0	99.0 ±0.25	26.2 ± 15.0	33.7 ± 37.3	50.7 ± 7.37	4.23 ± 5.36	208 ± 11.1	30.2 ± 13.02
3_SL1+2	61.4 ± 4.35	61.9 ±18.1	97.4 ±1.95	13.1 ± 8.10	11.5 ± 7.02	10.7 ± 1.53	10.2 ± 2.80	87.9 ± 6.31	25.0 ± 1.15
3_S2m	37.4 ± 7.63	5.03 ±2.22	40.4 ±7.23	6.50 ± 8.42	11.1 ± 6.93	10.3 ± 1.53	8.82 ± 4.95	54.4 ± 16.2	24.0 ± 1.83

Aliphatic	*Clomiphene citrate*			*qz2*			*trim*		
Sequence	*LB (%)*	*LOGSY factor (%)*	*CPMG variation (%)*	*LB (%)*	*LOGSY factor (%)*	*CPMG variation (%)*	*LB (%)*	*LOGSY factor (%)*	*CPMG variation (%)*
5_SL2+3	0.62 ± 21.7	2.75 ± 1.70	23.3 ± 11.0	-0.01 ± 9.84	2.85 ± 1.28	-3.85 ± 10.15	19.1 ± 3.31	49.4 ± 8.42	31.0± 7.21
ATTL	35.1 ± 26.9	2.42 ± 1.75	38.5 ± 8.81	4.09 ± 4.76	4.38 ± 2.13	1.57 ± 13.6	10.0 ± 4.83	5.15 ± 3.75	11.7 ± 5.50
PRF	68.7 ± 12.3	39.4 ± 0.10	86.2 ± 11.8	-3.21 ± 6.45	3.74 ± 2.19	-10.71 ± 14.1	13.9 ± 5.40	29.0 ± 11.3	14.0 ± 4.58
ORF7b	68.0 ± 4.89	40.4 ± 10.9	94.7 ± 5.31	-7.94 ± 5.76	6.35 ± 2.09	-18.0 ± 18.1	13.5 ± 4.26	103 ± 40.1	38.7 ± 4.73
3_SL1+2	70.7 ± 13.1	44.2 ± 11.2	94.5 ± 3.00	-2.14 ± 7.16	4.45 ± 1.98	-12.5 ± 12.9	14.0 ± 2.05	33.1 ± 1.48	18.3 ± 5.51
3_S2m	44.9 ± 12.5	3.03 ± 1.87	39.5 ± 8.38	1.30 ± 10.4	3.15 ± 1.79	-0.71 ± 12.6	9.33 ± 3.35	39.4 ± 8.65	15.7 ± 6.51

B

LB (%)/CSP (Hz)[a,b]

clomiphene citrate	5_SL2+3	ATTL	PRF	ORF7b	3_SL1+2	3_S2m
D	-1.34	15.9	81.7	67.6	61.4	37.4
C	-17.3	23.9	59.6	71.7	66.9	35.3
B	13.7	67.0	82.5	67.2	89.5	51.5
A	23.4	25.5	73.2	61.5	59.6	57.5

CPMG variation (%)[a]

clomiphene citrate	5_SL2+3	ATTL	PRF	ORF7b	3_SL1+2	3_S2m
D	29.0	41.4	99.0	97.5	99.1	40.4
C	14.9	40.8	77.1	92.9	91.5	40.8
B	26.7	38.3	93.9	96.5	97.0	38.8
A	36.0	34.3	96.5	96.3	99.3	37.1

LOGSY factor (%)[a]

clomiphene citrate	5_SL2+3	ATTL	PRFL	ORF7b	3_SL1+2	3_S2m
D	4.44	4.11	57.5	58.0	61.9	5.03
C	4.08	2.18	40.3	42.1	47.1	3.54
B	2.12	3.18	41.5	42.9	45.5	2.24
A	2.48	1.90	36.3	36.2	39.9	3.30

[a]LB and CPMG variations > 20%, LOGSY factors > 20% and CSP > 4 Hz are indicated in pink
[b]CSP values > 4 Hz are reported

LB (%)/CSP (Hz)[a,b]

qz2	5_SL2+3	ATTL	PRF	ORF7b	3_SL1+2	3_S2m
K	6.54	-5.00	1.11	9.52/5.7	5.71	-3.23
J	-6.27	12.1	-1.72	38.3/5.0	21.8	11.3
I	5.64	3.05	-0.20	30.9/4.5	11.8	11.5
F	-13.0	-1.84	-8.91	-14.4	-9.61	-12.7
G	3.46	8.16	1.78	-8.90	-3.71	4.32
E/B	8.30	7.56	3.04	-1.09	7.16	11.4
C/D	-0.32	2.52	-8.83	-10.7	-4.34	2.61
A	6.29	6.48	-3.66	-5.02	-2.06	4.87

CPMG variation (%)[a]

qz2	5_SL2+3	ATTL	PRF	ORF7b	3_SL1+2	3_S2m
K	3.01	10.5	14.6	58.5	8.91	11.6
J	5.88	9.03	11.4	45.5	11.1	9.16
I	4.89	9.63	13.2	48.3	11.7	10.3
F	-1.52	7.26	-0.60	-4.16	-4.16	6.38
G	-3.33	2.72	-7.24	-19.2	-11.4	-1.76
E/B	-11.8	-8.59	-18.2	-16.1	-19.5	-10.6
C/D	-2.36	3.98	-17.7	-34.1	-16.2	-1.93
A	3.62	7.14	-12.9	-33.2	-13.7	6.71

LOGSY factor (%)[a]

qz2	5_SL2+3	ATTL	PRF	ORF7b	3_SL1+2	3_S2m
K	4.04	14.4	11.4	17.9	3.38	18.7
J	7.22	5.22	42.7	76.3	16.0	9.42
I	6.13	10.3	13.3	7.00	15.1	5.19
F	2.30	3.39	3.69	6.18	3.95	2.81
G	1.79	6.53	2.32	6.45	4.70	1.92
E/B	4.57	2.78	2.11	5.54	3.76	6.11
C/D	3.84	6.82	7.49	9.65	7.61	3.32
A	1.74	2.39	3.10	3.94	2.25	1.60

[a]LB and CPMG area variations > 20%, LOGSY factors > 20% and CSP > 4 Hz are indicated in pink
[b]CSP values > 4 Hz are reported

LB (%)/CSP (Hz)[a,b]

trim	5_SL2+3	ATTL	PRF	ORF7b	3_SL1+2	3_S2m
E	16.7	23.3	-58.9	4.46	14.1/4.4	14.3
D	16.0	7.54	3.93	7.94	9.70	4.64
C	14.9	1.54	-39.2	-3.43	7.46	11.7
B	20.8	8.81	10.8	12.4	15.1	11.1
A	15.7	12.4	20.1	15.8	11.8	5.70

CPMG variation (%)[a]

trim	5_SL2+3	ATTL	PRF	ORF7b	3_SL1+2	3_S2m
E	38.9	-32.0	12.5	20.4	26.4	26.4
D	38.6	8.68	19.7	41.8	25.2	24.2
C	40.3	-36.9	9.72	18.3	23.6	22.2
B	36.9	17.6	18.9	44.0	23.8	22.1
A	32.9	8.20	13.0	34.9	18.5	15.8

LOGSY factor (%)[a]

trim	5_SL2+3	ATTL	PRF	ORF7b	3_SL1+2	3_S2m
E	135	3.98	88.8	196	90.5	38.2
D	126	4.51	46.0	218	92.6	70.5
C	131	4.56	16.7	210	80.8	54.5
B	43.4	2.50	21.0	132	34.2	33.3
A	55.3	7.80	37.0	75.4	32.1	45.5

[a]LB and CPMG area variations > 20%, LOGSY factors > 20% and CSP > 4 Hz are indicated in pink
[b]CSP values > 4 Hz are reported

Table S4. 3_S2m RNA interaction parameters of trim, clomiphene and qz2, measured by fluorescence intensity experiments.

compound[a]	K_d (µM) 0 mM NaCl	K_d (µM) 150 mM NaCl	K_d*-tRNA* (µM) 150 mM NaCl	specificity ratio 150 mM NaCl
trim	19.3 (13.1 - 29.1, 0.9331)	12.5 (8.48–18.5; 0.9325)	8.97 (6.04-11.9, 0.9305)	1.39
clomiphene citrate	36.0 (27.7 – 47.3, 0.9573)	33.2 (21.1 – 45.3; 0.9336)	16.8 (12.4-21.2, 0.9573)	1.99
qz2[b]	26.7 (21.4 - 33.6, 0.9778)	>50[c]	n/d	n/d

[a]For each compound, the table reports best-fit 3_S2m equilibrium dissociation constants, measured under two ionic conditions. At 150 mM NaCl, binding was evaluated in the absence (K_d) and presence (K_d-tRNA) of a 100-fold molar excess of tRNA. The specificity of the interaction was quantified with the K_d/K_d-tRNA ratio: interactions with specificity ratios close to 1 are specific, whereas those with ratios << 1 are unspecific. All experiments were repeated at least three times, and the 95% confidence intervals and R^2 coefficients are shown in parentheses. n/d: not determined.

[b]qz2 fluoresced at concentrations > 50 µM and the reported K_d value should be considered approximate.

[c]No clear transition was observed in this ionic condition.

Supplementary references

1. Prado, S.; Beltrán, M.; Coiras, M.; Bedoya, L.M.; Alcamí, J.; Gallego, J. Bioavailable inhibitors of HIV-1 RNA biogenesis identified through a Rev-based screen. *Biochem Pharmacol* **2016**, *107*, 14-28, doi:10.1016/j.bcp.2016.02.007.
2. Simba-Lahuasi, A.; Bedoya, L.M.; Alcami, J.; Gallego, J. Unpublished results. **2022**.
3. Martín-Villamil, M.; Sanmartín, I.; Moreno, A.; Gallego, J. Pharmacophore-based discovery of viral RNA conformational modulators. *Pharmaceuticals* **2022**, *15*, 748, doi:10.3390/ph15060748.
4. Prado, S.; Beltrán, M.; Moreno, A.; Bedoya, L.M.; Alcamí, J.; Gallego, J. A small-molecule inhibitor of HIV-1 Rev function detected by a diversity screen based on RRE-Rev interference. *Biochemical Pharmacology* **2018**, *156*, 68-77. doi:10.1016/j.bcp.2018.07.040.
